# Rationale for a Swedish cohort consortium

**DOI:** 10.1080/03009734.2018.1556754

**Published:** 2019-01-08

**Authors:** Johan Sundström, Cecilia Björkelund, Vilmantas Giedraitis, Per-Olof Hansson, Marieann Högman, Christer Janson, Ilona Koupil, Margareta Kristenson, Ylva Trolle Lagerros, Jerzy Leppert, Lars Lind, Lauren Lissner, Ingegerd Johansson, Jonas F. Ludvigsson, Peter M. Nilsson, Håkan Olsson, Nancy L. Pedersen, Andreas Rosenblad, Annika Rosengren, Sven Sandin, Tomas Snäckerström, Magnus Stenbeck, Stefan Söderberg, Elisabete Weiderpass, Anders Wanhainen, Patrik Wennberg, Isabel Fortier, Susanne Heller, Maria Storgärds, Bodil Svennblad

**Affiliations:** a Department of Medical Sciences, Uppsala University, Uppsala, Sweden;; b Uppsala Clinical Research Center (UCR), Uppsala, Sweden;; c Department of Public Health and Community Medicine/Primary Health Care, Institute of Medicine, Sahlgrenska Academy, University of Gothenburg, Gothenburg, Sweden;; d Department of Public Health and Caring Sciences, Uppsala University, Uppsala, Sweden;; e Department of Molecular and Clinical Medicine, Institute of Medicine, Sahlgrenska Academy, University of Gothenburg, Gothenburg, Sweden;; f Department of Public Health Sciences, Stockholm University, Stockholm, Sweden;; g Department of Public Health Sciences, Karolinska Institutet, Stockholm, Sweden;; h Department of Medical and Health Sciences, Division of Community Medicine, Linköping University, Linköping, Sweden;; i Department of Medicine, Unit of Clinical Epidemiology, Karolinska Institutet, Stockholm, Sweden;; j Department of Endocrinology, Metabolism and Diabetes, Karolinska University Hospital Huddinge, Huddinge, Sweden;; k Västerås Centre for Clinical Research, Uppsala University, Uppsala, Sweden;; l Department of Public Health and Community Medicine/Epidemiology and Social Medicine, Institute of Medicine, Sahlgrenska Academy, University of Gothenburg, Gothenburg, Sweden;; m Department of Odontology, School of Dentistry, Umeå University, Umeå, Sweden;; n Department of Medical Epidemiology and Biostatistics, Karolinska Institutet, Stockholm, Sweden;; o Department of Pediatrics, Örebro University Hospital, Örebro University, Örebro, Sweden;; p Department of Clinical Sciences, Skane University Hospital, Malmö, Lund University, Lund, Sweden;; q Department of Clinical Sciences, Cancer Epidemiology, Lund University, Lund, Sweden;; r Department of Psychiatry, Icahn School of Medicine at Mount Sinai, New York, NY, USA;; s Seaver Autism Center for Research and Treatment at Mount Sinai, New York, NY, USA;; t Department of Clinical Neuroscience, Karolinska Institutet, Stockholm, Sweden;; u Department of Public Health and Clinical Medicine, and Heart Center, Umeå University, Umeå, Sweden;; v Department of Research, Cancer Registry of Norway, Institute of Population-Based Cancer Research, Oslo, Norway;; w Genetic Epidemiology Group, Folkhälsan Research Center, Faculty of Medicine, Helsinki University, Helsinki, Finland;; x Department of Community Medicine, University of Tromsø, The Arctic University of Norway, Tromsø, Norway;; y Department of Surgical Sciences, Uppsala University, Uppsala, Sweden;; z Department of Public Health and Clinical Medicine, Family Medicine, Umeå University, Umeå, Sweden, and;; aa Research Institute of the McGill University Health Centre, Montreal, Canada

**Keywords:** Common infrastructure, epidemiological research, pilot study, rare outcomes, Swedish cohort consortium

## Abstract

We herein outline the rationale for a Swedish cohort consortium, aiming to facilitate greater use of Swedish cohorts for world-class research. Coordination of all Swedish prospective population-based cohorts in a common infrastructure would enable more precise research findings and facilitate research on rare exposures and outcomes, leading to better utilization of study participants’ data, better return of funders’ investments, and higher benefit to patients and populations. We motivate the proposed infrastructure partly by lessons learned from a pilot study encompassing data from 21 cohorts. We envisage a standing Swedish cohort consortium that would drive development of epidemiological research methods and strengthen the Swedish as well as international epidemiological competence, community, and competitiveness.

## Background

### United we stand, divided we fall

At the turn of the millennium, it was recognized that candidate gene association studies generated a large amount of non-replicable results. This rapidly led to a common understanding among genetic epidemiologists of the need for very large sample sizes in order to generate robust results. These insights seem not to have disseminated consistently to non-genetic epidemiology. Effectively, the scientific literature is flooded with underpowered studies, and it is becoming increasingly recognized that these studies cannot be replicated, casting doubts on the credibility of research results in general (1). Hence, there is an urgent need for data resources where research results can be replicated in an independent sample. Further, for optimized return on investment of taxpayers’ and other funders’ money and maximized benefit to patients and populations, it is essential that researchers other than those who originally assembled a database can use and re-use those data (2). These insights point, as the way forward, to increasing access to individual cohorts and leveraging integration of data across studies to obtain the statistical power required to answer contemporary research questions.

### Why another cohort consortium, and why in Sweden?

The recognition of the need to collaborate has led to development of consortia of cohorts in the last decade. Many Swedish population-based cohorts already participate in a multitude of international cohort consortia. Some consortia are defined by access in the participating cohorts to specific exposures (e.g. the Emerging Risk Factors Collaboration, the International Database on Ambulatory blood pressure in relation to Cardiovascular Outcomes, or Research on European Children and Adults born Preterm) (3–5); others are defined by access to specific outcomes (e.g. the European Prospective Investigation into Cancer and Nutrition) (6); while yet others include broad selections of cohorts with access to very general exposures (e.g. the NCD Risk Factor Collaboration) (7). Hence, these consortia are by design limited in terms of inclusion and exclusion criteria for the participating cohorts and data content.

Sweden is a country with unique opportunities for epidemiological research. Together, the governmental agencies National Board of Health and Welfare (SoS) and Statistics Sweden (SCB) host a multitude of individual-level data of great importance for epidemiological researchers (exemplified in Table 1 and on www.registerforskning.se). Using the 12-digit personal identity number, unique to all Swedish citizens, information from the registers can be uniquely linked (8).

**Table 1.** Examples of Swedish official registries.

Registry	Contents
Swedish Total Population Registry (14)	Place of residency; country of own and parents’ birth; marital status; date of death or emigration
Swedish Censuses	Socio-economic group; education; income; sick leave
Swedish National Insurance Agency	Sick leave; pensions
Swedish Education Registry	Highest education
Swedish 9th Grade Registry	Junior high school grades
Swedish Multi-Generation Registry (15)	Number of children and siblings; identity of parents if born after 1932 and alive in 1961
Swedish Medical Birth Registry (since 1973) (16)	Numbers of pregnancies and births; pregnancy outcomes
Swedish Prescribed Drug Registry (since 2005) (17)	Pharmacy-expedited drug prescriptions
Swedish Inpatient Registry (since 1964, with complete national coverage since 1987) (18)	Diagnoses of all hospitalizations; surgical and other procedures
Swedish Cancer Registry (since the 1950s) (19)	All cancer diagnoses
Swedish Cause-of-Death Registry (20)	Causes of death, including contributing factors
Swedish Out-Patient Registries (day-care surgery since 1997, all others since 2001)	All diagnoses. Hospital-based mandatory; primary care voluntary

Because most Swedish prospective population-based cohorts have access to outcomes collected in these registries in a structured way across the whole nation, Swedish cohorts have close to complete follow-up for all its participants except for those who emigrate. All events that are severe enough to result in e.g. death, an admission or visit to any hospital (diagnoses classified using the International Classification of Diseases [ICD] system), a surgical procedure (classified using the Nordic Medico-Statistical Committee Classification of Surgical Procedures [NCSP] system), or a filled drug prescription (classified using the Anatomical Therapeutic Chemical Classification [ATC] system) are collected in the same way in all cohorts. Hence, a cohort consortium consisting exclusively of Swedish prospective population-based cohorts will bring to the table a unique possibility to study uncommon exposures and outcomes, classified prospectively.

### Other prerequisites for success

The prospective population-based cohort study in a rich official registry setting—where a defined, prospectively examined group of people is followed over time based on a personal identity number for register linkages—is a highly valuable observational study design. Sweden has a large number of carefully collected population-based cohorts that have been followed for decades. We have ongoing recruitment into several high-quality cohorts. As mentioned, we have a variety of national socio-demographic and medical registries, covering the whole population since many decades. In addition, we have a large number of leading epidemiological researchers in the country, with a combined knowledge spanning most current research fields. The Swedish government has also recently provided strategic targeted funding to epidemiological research.

However, Swedish cohort research is poorly coordinated today. Many research projects are underpowered by using only one cohort at a time, leading to uncertain results with little benefit to patients and the public. Furthermore, rare diseases and exposures are impossible to study in individual cohorts due to lack of statistical power and are therefore discriminated. Hence, the stage is set for an initiative to unite all Swedish cohorts in an infrastructure for cohort collaboration.

## Pilot study

**Figure 1.**
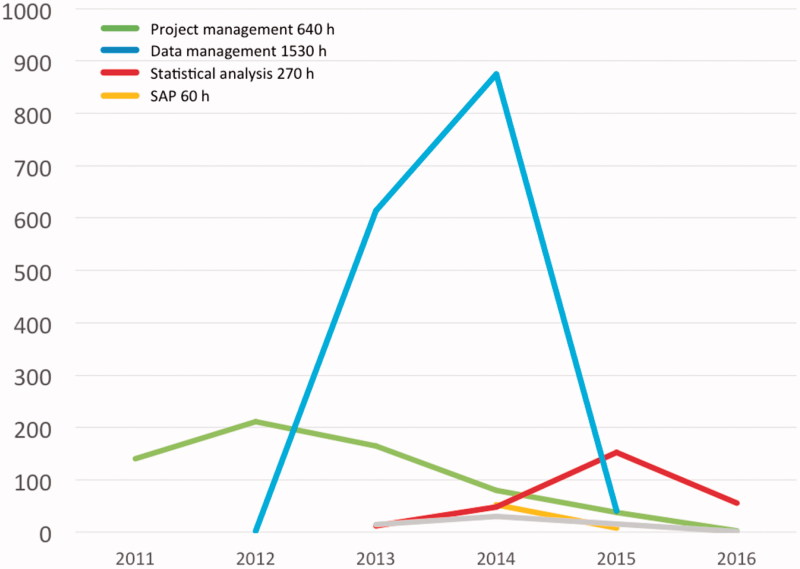
Time (h) spent on processes in the pilot study. SAP: statistical analysis plan.

In order to gain experience from administering a collaborative cohort project, we undertook a study to identify risk factors for subarachnoid haemorrhage (SAH) among more than one million cohort participants in 21 cohorts (9). SAH was chosen because it is comparatively rare and most cohorts would be too small to study this condition. Below, each step is first described and then commented. Eventually, the study was completed and presented at a scientific meeting (9). The time invested in the different processes is illustrated in Figure 1. The pilot study helped us chart and quantify the multitude of obstacles involved in cohort research in Sweden in general, and especially in a collaborative cohort project. The lessons learned from that project were essential for the development of the current proposal.

### Inclusion of cohorts

The first invitation to participate in the study was sent in February 2011 to 35 epidemiologists and data managers representing 29 Swedish cohorts. Because we did not know of all potential cohorts at the start, additional cohorts were invited and included until 2014. Ultimately, 21 cohorts participated in the study. Requirements for cohort data were stated in a research proposal, with very few inclusion (data on systolic and diastolic blood pressure and smoking status) and exclusion (previous SAH) criteria. Each cohort had to be enriched with data from official registries hosted by the National Board of Health and Welfare (SoS) or Statistics Sweden (SCB). As it turned out, all but five cohorts were enriched with such data.

Reasons for cohorts declining participation in the project were of different kinds; most often the cohort holders did not have the time, or data were deemed unreliable, incomplete, or unsuitable for the study by the cohort holders. Many cohorts required applications on their own forms, which was very time-consuming to comply with. Some cohorts did not have a steering group or any formal procedures for approval of projects, which tended to hamper communication.

### Ethics approval

**Figure 2.**
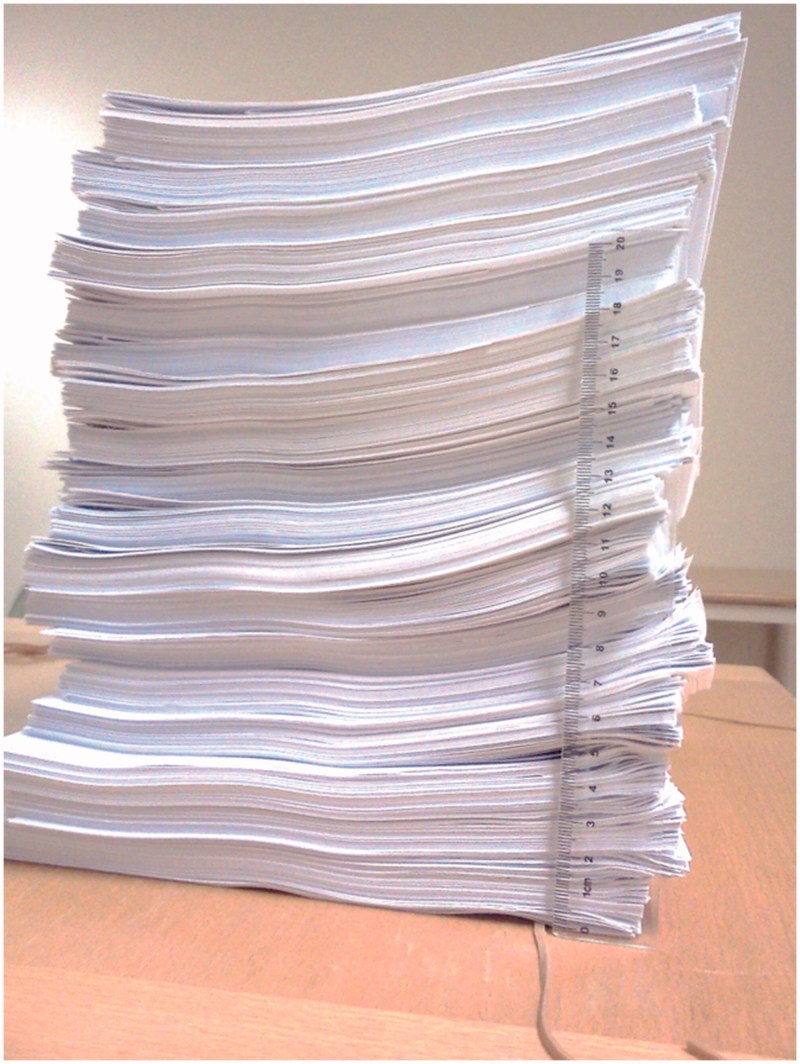
Resubmission to the ethics review board.

The primary application to the ethics review board was submitted when all cohorts were identified, and cohort holders had agreed to participate in the project. In the first round of comments, the ethics review board required details on inclusion criteria for all cohorts, descriptions of the data extraction procedure from each cohort, details on the information given to all participants in each cohort at enrolment and examples of informed consent forms, and descriptions of the informed consent procedures in each cohort. After submission of these complementary documents to the ethics review board (Figure 2), they found the information to the participants about the present study insufficient. The ethics review board required an advertisement in daily news press, with instructions on how to opt out of the project. This was done. The ethics review board approved the study after 6 months of correspondence.

None of the 949,683 cohort participants opted out of the study. One of the great benefits of cohort studies is the possibility to study many outcomes, including ones that were not identified at the time of the design of the cohort. The ethical and societal gains from that possibility (the eliminated need for conducting a new cohort study to answer every new research question) need to be better recognized and balanced against the limited potential harms.

### Data processing agreements

Personal data cannot legally be transferred between entities without a written agreement between the data controller (the sending part) and the data processor (the receiving part). Such agreements were set up between each cohort and Uppsala Clinical Research Center (UCR). It is the responsibility of the data controller to set up such agreements, but UCR did this as an extra service.

Many cohort representatives were entirely unfamiliar with the legal requirements. In some cases, UCR had not received copies of the signed agreements from the cohort representatives after two reminders, although UCR had signed them and received the data. In some cases, the cohorts had developed their own data transfer agreement forms, with unknown relationships to the legal data controlling entity (typically a university).

### Data transfer

The project used the UCR data transfer system, which complies with Swedish and EU data security laws (21, 22), and ensures that data are handled to the highest possible standard, with access restrictions and data encryption in transit and at rest. Contact between data managers for each cohort and the data manager at UCR was essential for secure data transfer in accordance with UCR standard operating procedures. In some cases, no cohort data manager was available, and the cohort holder or a research assistant made the data transfer.

It is of high importance to identify the data manager early in the process for most effective communication. One researcher sent the data using Hotmail, which led to a notification by the Quality Assurance team at UCR. A federated system such as DataSHIELD (11) without the need to physically send data would better safeguard participants’ integrity.

### Enrichment with registry data

Five of the 21 cohorts were not enriched with data from SCB and SoS at the time of the invitation to the study. For these five cohorts, the UCR project manager applied for selected variables from SoS and SCB. An application was written to SoS, and the same form was used for the application to SCB. The ethical approval together with a protocol synopsis were attached as well as a list of needed variables. The five cohort datasets were sent with personal identification numbers to SoS. SoS delivered the enriched datasets to SCB. SCB enriched the datasets further and sent them pseudonymized (without personal identification numbers) to the research group at UCR. This procedure was very time-consuming. One reason was that SoS and SCB appeared to have no direct communication between each other.

A federated system such as DataSHIELD (11) (and the system proposed in an accompanying article in this issue of the Journal) with participation of SoS and SCB, would be preferable for optimizing speed and integrity.

### Data harmonization

The final database required harmonization of a dozen variables. This harmonization is a crucial step in a project where the aim is to analyse pooled data. The harmonization work (processing study-specific data under a common format) was performed at UCR, and took a very long time for several reasons besides the difficulties related to data management and processing. The documentation for many of the variables (data dictionaries) was incomplete, or even non-existent, for many of the cohorts. The relevant staff at the cohorts was not always identified at the start, and finding the right person could be cumbersome. Even when the right person was identified, responses by mail or telephone were sometimes very slow. Preliminary results were presented to the cohort holders and data managers at a teleconference meeting. Misunderstandings of the research proposal were sorted out. Solving those issues was quite swift, and the database was considered final soon after the meeting.

Using dedicated tools such as Maelstrom (23) for data descriptions and dictionaries and for harmonization is likely to considerably speed up the process. Presenting descriptive analyses at a teleconference at an early stage is also likely to speed up harmonization—inspection of variable distributions stratified by cohorts gives opportunities for cohort representatives to identify mistakes or misunderstandings.

### Statistical analysis

A statistical analysis plan was presented in 2014, specifying that associations of risk factors with SAH should be analysed using Cox proportional hazards models, accounting for the clustered data structure using shared frailty models. During the analysis work, we decided to change to Poisson models instead. Reasons for this were that two time scales were of interest (calendar time and age) and that cohorts collected decades apart were difficult to model using Cox regression. Other peculiarities in the data observed at the time of analysis were that not all ages were present in all calendar periods; and problems with correctly identifying events and event-free time because registries used to capture the outcome did not cover all of Sweden until 1987, and because some cohorts with very long follow-up needed to account for several ICD code versions and had not done so initially.

It is a good idea to study descriptive statistics for each cohort in parallel with statistical analysis plan development and share those data with the cohorts, both in order to check harmonization of key variables and to make sure that the plan fits the data. Principles for missing data imputation should be determined at the outset, but may need to be refined once data are in. Good communication between principal investigator/statistician/data manager is important.

## Proposal

We have led an initiative for a national collaborative infrastructure, the *Swedish Cohort Consortium* (*Cohorts.se*). The full proposal can be found on www.cohorts.se, with contributing cohorts and researchers listed on https://snd.gu.se/sv/catalogue/keyword/cohortsse; Table 2 provides a condensed outline.

**Table 2.** Proposal for a Swedish Cohort Consortium (Cohorts.se).

Component	Description
Data management	Some Swedish cohorts are kept at the highest possible standard, with secure storage of data in modern data formats, including backups, controlled data access, and access logs. Many also have curated data with complete and documented procedures for data curation as a result of e.g. logical checks, monitoring, adjudication, or outlier detection; and with data curation traceable through an auditing feature in the data warehouse. Unfortunately, many valuable cohorts are kept under suboptimal circumstances in some or all of these respects. Sharing experiences and tools for efficient data management among cohort owners is an important feature of a cohort infrastructure. The Maelstrom Research offers guidelines (23) and a suite of open source software (24) (Opal, Onyx, Mica, and Agate) to support management of cohort data. Optimally, all cohorts in the infrastructure would run a local instance of Opal in conjunction with their other software stack (24). Opal can be used to achieve data management and harmonization. It is integrated with R, and, using the DataSHIELD approach, such an infrastructure could support advanced statistical data analysis across cohorts without having to share or disclose individual-level data (11).
Cataloguing	Cohort steering groups hold detailed knowledge and documentation on the cohorts’ design, sampling procedures, data collection methods, losses to follow-up, data quality, and other cumulative knowledge about the data gathered through a multitude of research projects. Given the complexity in the structure of the existing datasets and the rich content of the data, the experience of the cohort holders is invaluable for effective use of the data. This experience needs to be condensed into a useful metadata repository. In *Cohorts.se*, the Maelstrom approach for study and variables cataloguing is proposed. A key activity in joint cohort projects is searching for harmonization potential among cohorts with similar data. A prerequisite for this is a metadata catalogue containing structured details about the cohort design and the variables and samples collected in the cohort study. In *Cohorts.se*, it is proposed to use the Mica software offered by Maelstrom. Mica is a software application for web portals for individual cohorts or for consortia. When used in conjunction with the Maelstrom component Opal, for data management, Mica also allows authenticated users to perform distributed queries on the content of study databases hosted on remote servers and retrieve summary statistics (24). A pilot catalogue including 6 studies and 10,000 variables is accessible on www.maelstrom-research.org/mica/network/cohorts.se
Enrichment	In order to stay relevant, epidemiology needs collection of new cohorts, and re-investigation and expansion of existing cohorts. *Cohorts.se* proposes to customize and use the Maelstrom’s Onyx software to support high-quality collection of new cohorts. Onyx is a web-based application that manages participant logistics at cohort assessment clinics, including appointment management, controlling stage availability and dependencies, consents, questionnaires, sample collection (barcode scanning), linkage to sensory equipment such as ECGs or scales, producing personalized reports for participants, and exporting encrypted data to multiple destinations. In addition, *Cohorts.se* will aid in variable selection for new investigations of cohorts, guided by knowledge of usefulness of existing variable lists in other cohorts in *Cohorts.se. Cohorts.se* is also proposed to provide standardized integration of commonly used data sources like public national registries.
Project management	For researchers to conduct projects using the consortium, some information needs to be readily available, including cohort metadata, variable lists, definitions of variables, and standardized application forms for research proposals. We propose that *Cohorts.se* provides basic support for submission of research proposals, coordinates decisions to join a collaborative project, coordinates data access, provides statistical support as needed, and facilitates critical steps in the publication process. Further, *Cohorts.se* should facilitate compliance with legal and ethical requirements, and ensure complete anonymization of data before delivery to researchers, facilitate meta-analysis or, where relevant, use federated analysis such as DataSHIELD.
Harmonization	In order to make joint analyses, variables from different cohorts need to be measured on the same scale. Using the Maelstrom approach, harmonization of variables is facilitated and documented in stored scripts (25). Harmonized data can then be analysed within the Maelstrom system, or exported for analysis on other analysis platforms. The harmonization process is unique for each research project, but parts will be re-usable in subsequent projects. These parts will grow over time with shorter and shorter time needed for reprocessing.
Power calculations	*Cohorts.se* should be able to provide data useful for statistical power calculations for all cohorts storing data on an Opal server connected to Mica. At the moment, user-friendly tools for determining adequate sample size for more complex analyses that include clustered data, family designs, or multiple interactive effects are not readily available. Power calculations require detailed knowledge about the structure and contents of data in specific cohorts, including information on missing data for combinations of variables. Assuring that the existing data are adequate for a proposed analysis will likely be a common question.
Statistical analysis	Analysing harmonized data from different cohorts today often means transferring and pooling of individual data into a single large database, but that practice may be infeasible because of time, ethical, or legal issues. DataSHIELD offers a possibility to analyse data as if they were pooled, though still stored at local Opal servers behind firewalls (11). *Cohorts.se* can set up analysis servers at several participating universities, permitting different analyses of the joint cohort data from multiple access points.
Local cohort operations	The backbone of *Cohorts.se* is the participating cohorts and all persons involved in the management of the cohorts. For a successful infrastructure, incentives must be developed for the cohort holders to keep their cohorts safely stored in modern formats on adequate hardware, accessible for research projects, up to date in the *Cohorts.se* catalogue, sufficiently staffed with data management personnel, and governed by a functioning steering committee or other body. Within *Cohorts.se*, a charter with recommendations of levels of governance, maintenance, data storage, and data curation will be proposed.

The long-term scientific goal is to facilitate greater use of Swedish cohorts for world-leading research; excellence would mainly be achieved by making cohort data discoverable and more accessible and supporting collaborations between cohorts.

Important components of such an infrastructure would build on open access and open science and involve data curation and management, cataloguing study designs and variables content, developing common procedures for access to data, harmonizing variables to support research projects, linking cohorts and official registries using novel techniques, achieving statistical analysis including a method for distributed data analysis eliminating the need to send data between cohorts, and implementing collaborative web interfaces for researchers, data managers, statisticians, and publication managers.

The proposed undertaking is not trivial. Even with appropriate approaches such as these, the terminologies, procedures, technologies, and methods used vary markedly between cohorts. Because of this complexity and the heterogeneity of information collected from pre-existing cohorts, integration of the information presents major challenges. Achieving scientifically valid harmonization requires secure data environments and specialized expertise and resources. Our initiative aims to fill this need.

## Ethical considerations

Ethical, legal, and social implications of collaborative cohort analysis arise from the fact that cohorts are initiated with different goals, and that participants in different cohorts therefore will have been given different information about the intended use of the data. The consent procedures may vary widely across cohorts. Permission from an ethics review board is a valid proxy for consent from the cohort participants when applied to secondary analyses of the data (10), and handling of all collaborative projects in the infrastructure at an ethics review board experienced in such research, such as the procedure in the UK Biobank, would be a great advantage.

Other ethical issues may result from an increased accessibility of the cohorts to the research community, including risks of breaches of security and privacy. *Cohorts.se* aims to develop appropriate means of data access that ensure privacy and secure data handling. In order to protect the privacy of individuals, but at the same time utilize the strength that some individuals participate in multiple cohorts, a novel secure technique has been proposed for joining cohorts using the personal identity number (see accompanying paper by Snäckerström and Johansen in this issue of the Journal). Use of DataSHIELD (11) also minimizes the risk.

The main ethical gains from facilitating collaborative cohort analysis include benefits to patients and populations in the form of more precise, timely, and reliable research findings due to better-powered studies, and research findings that would be impossible to obtain without collaborative analysis. Ethical gains from increasing the accessibility to and use of cohort data are that study participants’ data are better utilized and their donation therefore becomes more valuable, with higher benefit to patients and populations.

Other ethical advantages may include safer management of sensitive person data and higher equality in access to necessary support functions. Today, the management of some cohorts is very vulnerable, with insecure backup routines and key knowledge about the cohort maintained by single persons. In *Cohorts.se*, data management will be significantly improved compared to the standards in place at the weakest environments today. We propose common data and material transfer agreements for all cohorts, saving time and ensuring that all cohorts are treated equally. We also propose transparent and clearly stated common rules for liabilities, access rights, and limits of use based on international charters for sharing and access to data.

## Potential gains to society

### Less waste in research

*Cohorts.se* will facilitate modern data curation and management, which will allow us to increase the level of data security, ensuring future-proof storage of valuable Swedish cohorts. There is a clear threat of total oblivion for older cohorts with low to minimal use and vulnerable management structures. *Cohorts.se* will catalogue all cohorts in a detailed and structured way, which will facilitate use of valuable cohort data that are known only to a smaller circle of researchers today and therefore under-used. A pilot catalogue including six studies and 10,000 variables is accessible on www.maelstrom-research.org/mica/network/cohorts.se.

*Cohorts.se* will reduce the time wasted waiting for results. As an example, the 14,000,000 person-years of follow-up in the pilot study of this infrastructure (9) would take UK Biobank (500,000 screened subjects) 28 years to acquire. This means that *Cohorts.se* will allow world-leading research that may be impossible to achieve elsewhere today.

A common infrastructure for all cohorts can facilitate access to data about the cohort participants held by Statistics Sweden and the National Board of Health and Welfare, and potentially reduce the very long (currently up to 2 years) waiting times for data at these governmental agencies.

Combining multiple cohorts permits better-powered solutions for any research question that is today explored in single cohorts, generating more precise results. But it is especially valuable as it permits timely and adequately powered research on rare diseases, rare exposures, and extreme levels of exposures.

### Building competence, community, and competitiveness

*Cohorts.se* will provide collaborative interfaces for researchers, data managers, and biostatisticians, including national meetings and educational efforts. We have good reason to believe that this will drive development of epidemiological research methods, increase the quality of research projects, maximize the prospects of getting research projects funded by international funding agencies, and in the long run secure excellent research environments and influx and retention of excellent researchers. This has been the case in Norway, where a structured collaboration between cohorts over two decades has played an important role in developing epidemiological research environments and projects and facilitating cohort collaborations (12). Further support for the forecast that a cohort collaboration infrastructure will drive research excellence comes from the Emerging Risk Factors Collaboration. This task has led to a very strong development of cohort research methods, the legacy of which includes important publications in leading medical journals, a large suite of statistical software developments, shared freely on their website (13), as well as personal experience, competence, and networks among the leading researchers.

This proposal has been developed at two national workshops among cohort researchers taking part in the pilot study of *Cohorts.se*, and subsequently in writing groups composed of researchers from all parts of Sweden. Participants in these activities have reported the networking with new acquaintances with similar interests as very fruitful. The popularity of this proposal is also reflected in the fact that since the initiation of the pilot study the number of cohorts in the initiative has more than doubled, with a total of more than 40 cohorts at seven universities participating in the proposal. *Cohorts.se* aims to eventually embrace all interested prospective population-based Swedish cohorts.

The long-term strategic importance of *Cohorts.se* for the Swedish medical research community is great. It will add lasting value to existing cohorts, registries, and researcher groups, providing an opportunity for Sweden to obtain a leading position in epidemiology and further attract leading international researchers. It can facilitate Swedish participation and leadership in international cohort collaborations, such as those in Table 3. The importance to the pharmaceutical industry of uncovering the pathophysiology of uncommon diseases may also be vital.

**Table 3.** International infrastructures with which collaboration may be sought.

Name	Website
The Asia-Pacific Cohort Studies Collaboration	www.apcsc.net
Emerging Risk Factors Collaboration	www.phpc.cam.ac.uk/ceu/research/erfc
Prospective Studies Collaboration	www.ctsu.ox.ac.uk/research/meta-studies/psc/psc-website
Monica Risk, Genetics, Archiving and Monograph (MORGAM)	www.thl.fi/morgam
Biomarker for Cardiovascular Risk Assessment in Europe (BiomarCaRE)	www.biomarcare.eu
National Cancer Institute (NCI) Cohort Consortium	http://epi.grants.cancer.gov/Consortia/cohort.html
European Cohort Consortium (follow-up to BBMRI-LPC)	www.bbmri-lpc.org
Research on European Children and Adults born Preterm (RECAP)	https://recap-preterm.eu/

The Maelstrom software suite (23) is used for several of these collaborations, including the NCI Cohort Consortium, BBMRI-LPC, and RECAP. Using the same suite of tools for *Cohorts.se* will facilitate collaboration with those international consortia. Some Swedish cohorts are already catalogued in Maelstrom, including ULSAM, EpiHealth, LifeGene and TwinGene. The continuation of the BBMRI-LPC is uncertain, but there are initiatives to transform it into a European Cohort Consortium, which will most likely be open for inclusion of more Swedish cohorts. Some of the other networks that use Maelstrom are described on https://www.maelstrom-research.org.

## Conclusion

In sum, coordination of all Swedish prospective population-based cohorts in a common infrastructure would enable more precise research findings and facilitate research on rare exposures and outcomes, leading to better utilization of study participants’ data, better return of funders’ investments, and greater benefit to patients and populations. We envisage a strong standing Swedish cohort consortium that would drive development of epidemiological research methods, and strengthen the Swedish epidemiological competence, community, and competitiveness.

## Note on corresponding author

Johan Sundström is Professor of Epidemiology at Uppsala University, and a cardiologist at Uppsala University Hospital.
